# Supplementary material for: Emergence of transformation-tolerant representations of visual objects in rat lateral extrastriate cortex
Source: eLife. 2017 Apr 11;6:e22794. doi: 10.7554/eLife.22794 (PMC5388540; doi:10.7554/eLife.22794)
Supplement: Figure 1—source data 1. — DOI: http://dx.doi.org/10.7554/eLife.22794.004 [file elife-22794-fig1-data1.docx]

| **Animal** | **Number of neurons recorded in each area** | | | |
| --- | --- | --- | --- | --- |
|  | **V1** | **LM** | **LI** | **LL** |
|  |  |  |  |  |
| Rat #1 | 0 | 37 | 8 | 0 |
| Rat #2 | 0 | 15 | 0 | 0 |
| Rat #3 | 33 | 0 | 0 | 0 |
| Rat #4 | 0 | 0 | 12 | 11 |
| Rat #5 | 27 | 0 | 0 | 0 |
| Rat #6 | 0 | 0 | 10 | 6 |
| Rat #7 | 0 | 10 | 23 | 12 |
| Rat #8 | 0 | 0 | 36 | 24 |
| Rat #9 | 0 | 0 | 11 | 0 |
| Rat #10 | 3 | 12 | 12 | 4 |
| Rat #11 | 60 | 0 | 0 | 0 |
| Rat #12 | 0 | 23 | 13 | 0 |
| Rat #13 | 26 | 0 | 0 | 0 |
| Rat #14 | 0 | 0 | 14 | 1 |
| Rat #15 | 0 | 0 | 5 | 1 |
| Rat #16 | 0 | 3 | 12 | 16 |
| Rat #17 | 19 | 0 | 0 | 0 |
| Rat #18 | 0 | 20 | 16 | 0 |
| Rat #19 | 1 | 8 | 0 | 0 |
| Rat #20 | 0 | 0 | 1 | 1 |
| Rat #21 | 0 | 0 | 23 | 27 |
| Rat #22 | 37 | 0 | 0 | 0 |
| Rat #23 | 22 | 0 | 0 | 0 |
| Rat #24 | 0 | 2 | 8 | 0 |
| Rat #25 | 0 | 1 | 55 | 47 |
| Rat #26 | 0 | 0 | 1 | 2 |
|  |  |  |  |  |
| **Total** | **228** | **131** | **260** | **152** |
